# The All Saints Sisters' Home for Trained Nurses

**Published:** 1887-01-29

**Authors:** 


### A Noble Profession: Nursing the Sick.

### THE ALL SAINTS SISTERS' HOME FOR TRAINED NURSES.

By Miss Simpson.

It has been suggested that an account of this work might prove of value in more senses than one, and now, when it is crippled for want of funds and in pressing need of immediate aid, I venture, on behalf of the Sisters of All Saints to present to the public this, the first statement, although the Home has been quietly working from its own resources for some years.

In 1876, the Superior of All Saints Home, 82, Margaret Street, with the kind assistance of a few friends, took a house (3, Fitzroy Street), for a Home for Trained Nurses, who could be sent out to nurse either rich or poor in their own homes, always at a reasonable rate of payment, often free of charge. Suffering and sickness always can claim the ready help of the nurses, without distinction of church, party, or class.

The Home also provides a comfortable residence for the nurses when not engaged, or when ill and requiring rest. Provision, with a small pension, is also made for those nurses who from illness or old age are incapacitated for further service.

The Sisters hoped to make their Nurses' Home self-supporting; they find it impossible, the nurses' earnings barely covering the expenses. The gift of *free* nursing to those unable—and there are many—to pay a nurse for her service will have to be given up. It is the noblest part of their work, and the Sisters are very reluctant to withdraw from it; but the endeavour to carry it on without *outside* help has resulted in opening the new year with a heavy debt. The All Saints Sisters have striven very bravely to help themselves. In closing my little sketch of their work, may I remind my readers how very heavily the carrying out of a benevolent scheme sometimes rests upon the shoulders of the originators, how sorely the burden of debt presses? In their first zeal for a new work, many will help it forward, who by-and-bye grow weary, or forget to help any longer, but on *some one* must rest the anxious thought, the responsibility of carrying it on; and all honour to the brave hearts who, for the sake of suffering humanity, are striving under great difficulty to bring a good undertaking to a successful issue.

Subscriptions and donations will be gratefully received by the Sister Superior, 3, Fitzroy Street, Fitzroy Square, or paid into the account of the All Saints Nurses' Home,—Sir S. Scott, Bart., and Co., 1 Cavendish Square.

